# Predicting the complexity and mortality of polytrauma patients with machine learning models

**DOI:** 10.1038/s41598-024-58830-0

**Published:** 2024-04-09

**Authors:** Meiqi Yu, Shen Wang, Kai He, Fei Teng, Jin Deng, Shuhang Guo, Xiaofeng Yin, Qingguo Lu, Wanjun Gu

**Affiliations:** 1 School of Artificial Intelligence and Information Technology, Nanjing University of Chinese Medicine, Nanjing, 210023 Jiangsu China; 2 Jiangsu Province Engineering Research Center of TCM Intelligence Health Service, Nanjing University of Chinese Medicine, Nanjing, 210023 Jiangsu China; 3 Department of Orthopedics and Traumatology, Peking University People’s Hospital, Beijing, 100044 China; 4 Trauma Center, Pizhou People’s Hospital, Xuzhou, 221300 Jiangsu China; 5 Collaborative Innovation Center of Jiangsu Province of Cancer Prevention and Treatment of Chinese Medicine, Nanjing University of Chinese Medicine, Nanjing, 210023 Jiangsu China; 6 Key Laboratory of Trauma and Neural Regeneration (Peking University), Ministry of Education, 100044 Beijing, China; 7 National Center for Trauma Medicine, 100044 Beijing, China

**Keywords:** Machine learning models, Polytrauma mortality, Polytrauma complexity, Prognosis, Trauma, Outcomes research

## Abstract

We aim to develop machine learning (ML) models for predicting the complexity and mortality of polytrauma patients using clinical features, including physician diagnoses and physiological data. We conducted a retrospective analysis of a cohort comprising 756 polytrauma patients admitted to the intensive care unit (ICU) at *Pizhou* People’s Hospital Trauma Center, Jiangsu, China between 2020 and 2022. Clinical parameters encompassed demographics, vital signs, laboratory values, clinical scores and physician diagnoses. The two primary outcomes considered were mortality and complexity. We developed ML models to predict polytrauma mortality or complexity using four ML algorithms, including Support Vector Machine (SVM), Random Forest (RF), Artificial Neural Network (ANN) and eXtreme Gradient Boosting (XGBoost). We assessed the models’ performance and compared the optimal ML model against three existing trauma evaluation scores, including Injury Severity Score (ISS), Trauma Index (TI) and Glasgow Coma Scale (GCS). In addition, we identified several important clinical predictors that made contributions to the prognostic models. The XGBoost-based polytrauma mortality prediction model demonstrated a predictive ability with an accuracy of 90% and an *F*-score of 88%, outperforming SVM, RF and ANN models. In comparison to conventional scoring systems, the XGBoost model had substantial improvements in predicting the mortality of polytrauma patients. External validation yielded strong stability and generalization with an accuracy of up to 91% and an AUC of 82%. To predict polytrauma complexity, the XGBoost model maintained its performance over other models and scoring systems with good calibration and discrimination abilities. Feature importance analysis highlighted several clinical predictors of polytrauma complexity and mortality, such as Intracranial hematoma (ICH). Leveraging ML algorithms in polytrauma care can enhance the prognostic estimation of polytrauma patients. This approach may have potential value in the management of polytrauma patients.

## Introduction

Trauma represents a leading global cause of mortality, claiming nearly 5.8 million lives annually^1^. Among different traumatic conditions, polytrauma stands out not only as the most prevalent in ICU but also as the highest mortality rate^2^. Polytrauma arises from simultaneous damage to various organs and systems of the human body due to external forces, typically caused by incidents such as traffic accidents, accidental injuries, or natural disasters^3,4^. The primary reasons lie in patients experiencing multiple types of injuries concurrently, including trauma, fractures, and visceral damage^5^. The consequences of polytrauma may include organ failure, infections, hemorrhagic shock, and the need for physical and psychological rehabilitation^6^. This complexity presents a significant challenge in the treatment of polytrauma patients, requiring efficient management in the emergency phase, surgical repair of damaged organs and systems, and the implementation of a comprehensive rehabilitation plan. To reduce the high mortality and morbidity rates in the post-traumatic course, early “preventive” interventions are necessary^5^. Therefore, effective treatment of polytrauma hinges on the early detection and timely management of those life-threatening injuries^7^.

However, the clinical presentation and its severity in polytrauma patients manifest complex variations at ICU admission. This complexity poses great challenges for clinicians in precisely evaluating and predicting the prognosis of these patients^8^. In current practices, clinicians often rely on some injury scales or scoring systems, including Injury Severity Score (ISS)^9^, Trauma Index (TI)^10^, and Glasgow Coma Scale (GCS)^11^. These scoring systems calculate the risk scores based on some clinical indicators such as injury locations and patients' consciousness levels. While these scoring systems offer some assistance, their criteria predominantly depend on the expertise of clinicians, making them susceptible to subjective influences and lacking objective and precise indicators^11^. Moreover, these scoring systems often overlook a wealth of trauma-related clinical physiological data.

In contrast, ML techniques can process a huge amount of intricate data and perform predictive analyses^12^. In recent years, ML models have been applied in analyzing large clinical data, providing new opportunities in addressing some problems of disease diagnosis and treatment^13–15^. For instance, in the context of sepsis, Islam et al*.*^14^ developed a ML model that incorporated physiological information to enhance the identification and prognosis assessment of sepsis patients. In the battle against COVID-19, Wang et al*.*^15^ employed a deep learning model to analyze chest CT images, facilitating the prompt screening of suspected COVID-19 cases in fever clinics and expediting diagnosis and isolation measures. Additionally, ML technology holds promise for optimizing stroke treatment, such as recommending the most suitable endovascular surgery or drug treatment regimen based on individual patient characteristics and imaging data^13^. These applications demonstrate the potential and value of ML models in clinical settings.

With the standardization of clinical trauma data, ML models have gained many attentions in trauma management as well^16–21^. For example, Gorczyca et al*.*^17^ employed logistic regression, random forests, gradient boosting machines, and feedforward neural networks to construct an ensemble learning model for risk prediction, uncovering complex relationships in trauma data. Hsu et al*.*^18^ used artificial neural networks to predict outcomes for patients with moderate to severe head injuries, while Eftekhar et al*.*^16^ built and compared artificial neural network and logistic regression models for predicting mortality after head trauma based on clinical parameters. Yu et al*.*^21^ and Ma et al*.*^20^ applied statistical methods to examine the predictive value of polytrauma severity scores in patient outcomes. Lin et al*.*^19^ employed univariate and multivariate logistic regression to identify the optimal trauma scoring method and explore factors associated with mortality in polytrauma patients. These studies have predominantly utilized ML and statistical methods to construct predictive models for trauma patient mortality or severity. However, these studies have primarily relied on demographics, trauma scores, and physiological indicators as predictive variables, often overlooked clinical physician diagnoses for the outcome prediction of trauma patients.

In order to overcome limitations in previous prognostic models of polytrauma, we developed ML models to predict the mortality and complexity for polytrauma patients upon hospital admission using several trauma-related physiological indicators and clinical diagnosis data (Supplementary Figure S1). We benchmarked various ML algorithms and identified the best-performing ML model based on its overall performance. We also explored the model's interpretability by analyzing feature importance. Finally, we compared our model with those commonly used scoring systems to assess whether our model surpasses traditional scoring systems in performance.

## Materials and methods

### Data collection

**Figure 1:**
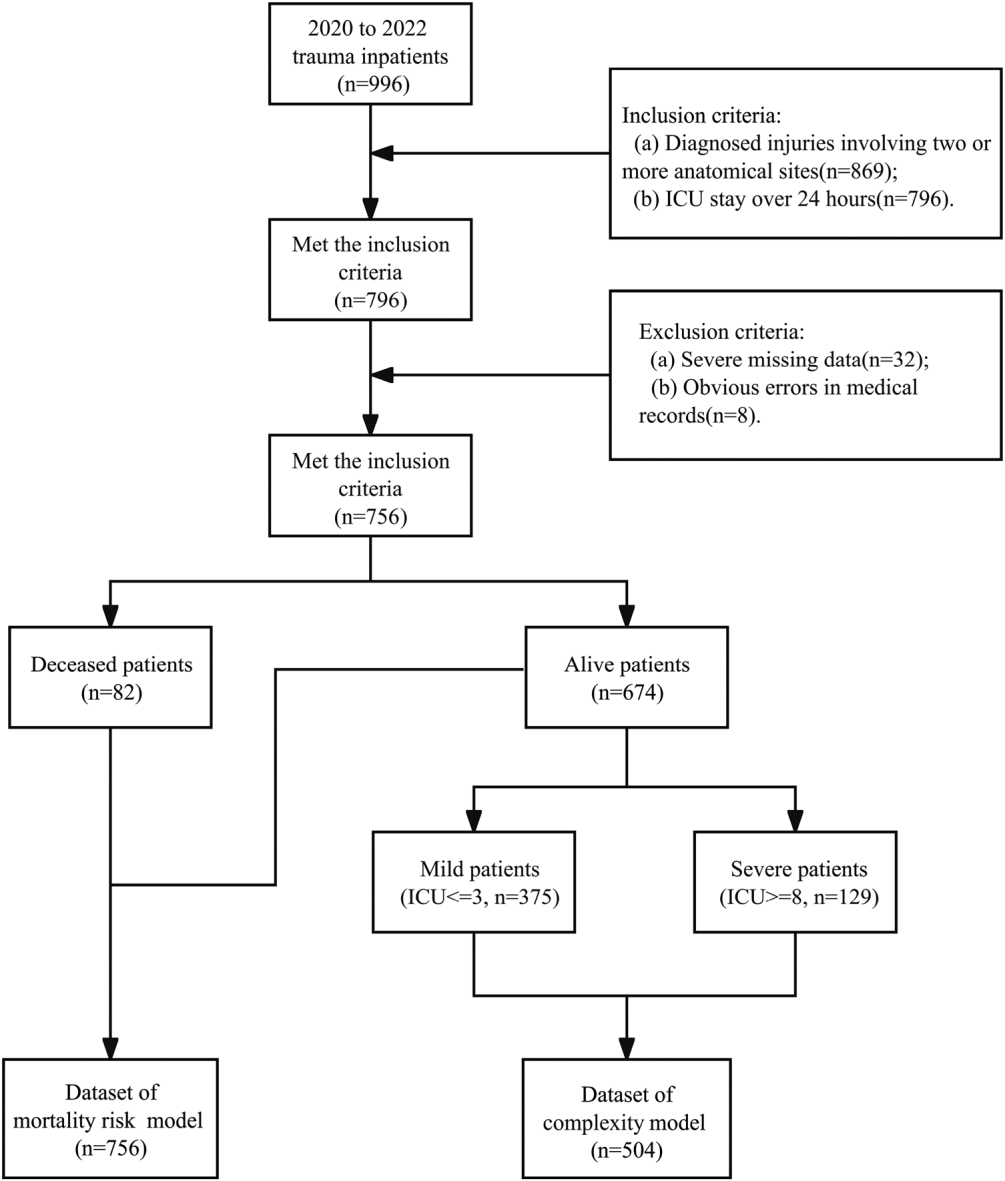
Workflow of data collection and processing. First, a total of 996 patients diagnosed with polytrauma before or upon admission were initially enrolled. Next, patients with ICU stays of more than 24 h and those with injuries involving two or more anatomical sites were included. Then, patients with severe missing data and obvious data errors were excluded, leaving 756 patients (674 survivors and 82 deceased) for model construction. All 756 patients were used to develop and validate the mortality risk model of polytrauma patients. In order to classify the complexity of polytrauma patients, we classified all 674 survived patients into mild, moderate and severe groups. Specifically, 375 polytrauma patients with ICU stays of 3 days or less were grouped into the mild group, while 129 patients with ICU stays of 8 days or more were grouped into the severe group. This resulted in a final dataset of 504 patients for the construction of polytrauma complexity model.

We retrospectively collected clinical data from trauma patients admitted to ICU at *Pizhou* People’s Hospital between January 2020 and December 2022. To avoid misdiagnosis and missed diagnosis, the management procedure of polytrauma patients in *Pizhou* People’s Hospital is to recruit them in the ICU at their first presentation, undergo necessary examination and monitoring, and then refer to different disciplines according to their trauma locations and severity. A total of 996 patients diagnosed with trauma were initially enrolled (Fig. 1). The collected characters encompassed demographic information, vital signs, records of surgical procedures during hospitalization, laboratory test results, and clinical diagnoses by physicians. 70 features were compiled in total, which were listed in Supplementary Table S1. To focus on polytrauma patients, we filtered 796 patients with injuries involving two or more anatomical sites and with ICU stays of more than 24 h (Fig. 1). To ensure data quality, patients with the missing data or obviously low quality data were excluded, resulting in 756 patients (674 alive and 82 deceased) for model development. Among them, the male-to-female ratio was 1.53:1, and the average age was 54.96 years. This study and its protocol were approved by the Ethics Committee of *Pizhou* People's Hospital. All data used in this study were desensitized, and Informed consent is not required for the use of such data. In addition, all analysis were carried out in accordance with the relevant guidelines and regulations, which was complied with the ethical requirements of China.

### Data preprocessing

To enhance data usability, we performed data preprocessing, including data cleaning, transformation and standardization on the raw dataset. We extracted those text features that are relevant to polytrauma from the primary diagnosis at admission, including various clinical diagnoses by physicians. To refine these primary diagnosis features, we first removed irrelevant diseases and invalid diagnostic information. Next, we categorized the filtered diagnosis text by different body regions, such as fractures, hematomas, dislocations, and injuries to various body parts (e.g., brain, cervical spine, thoracic spine, lumbar spine, joints, etc.). Supplementary Table S2 provides an overview of the categorized diagnostic features and their corresponding clinical diagnoses. These location-based features were then vectorized. In addition, as the raw dataset contained some missing values, we employed Multivariate Imputation by Chained Equations with random forest (MICE Forest)^22^ to impute these missing values. Finally, we standardized and normalized the imputed dataset to facilitate subsequent ML modeling. For standardization, StandardScaler^23^ was used to standardize features by removing the mean and scaling to unit variance. For normalization, MinMaxScaler^24^ was used to transform features by scaling each feature between 0 and 1. After data preprocessing, a final dataset with 70 clinical parameters was used as the input data of all ML models in model development.

### Model development

**Figure 2:**
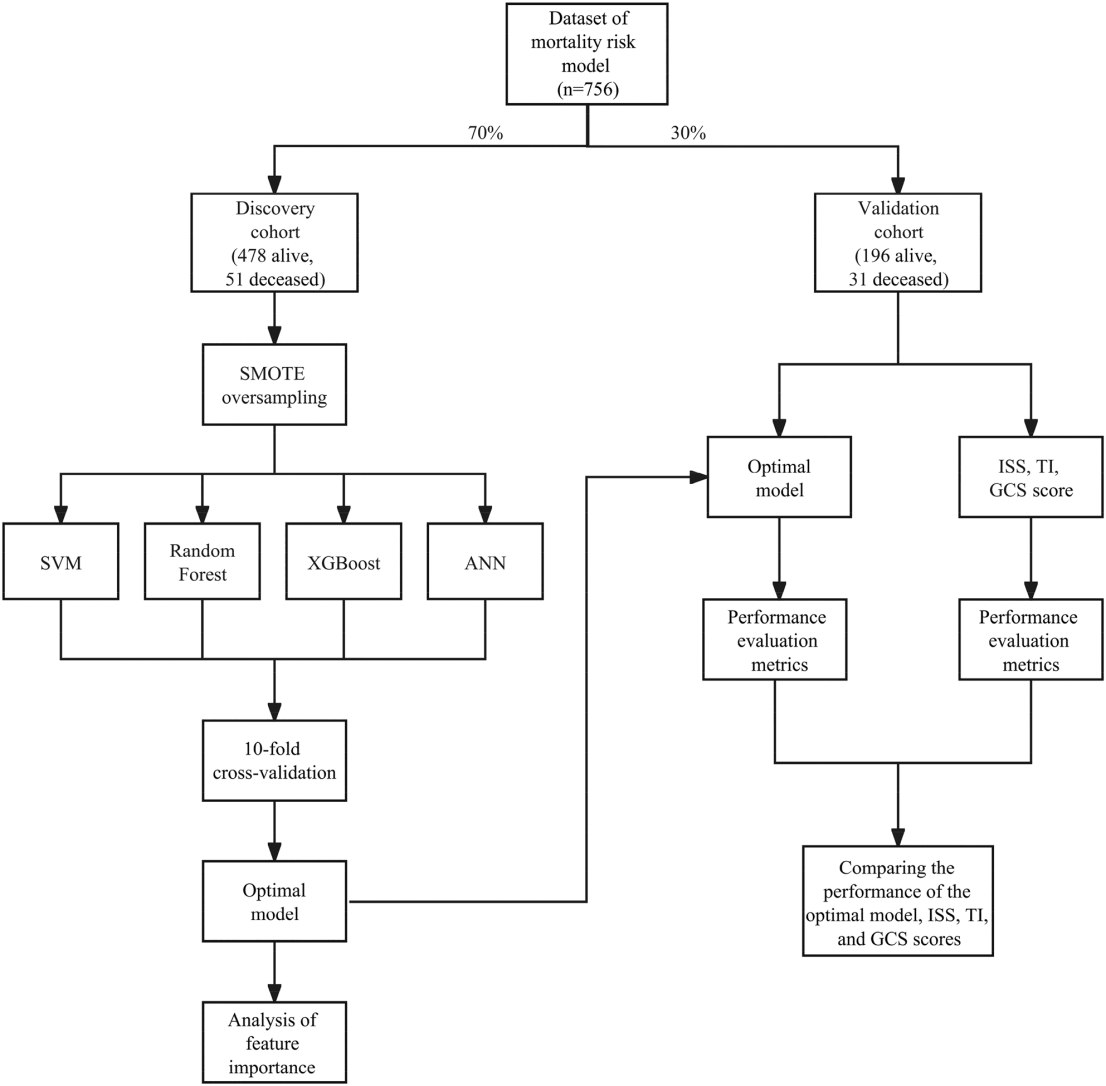
The construction of polytrauma mortality model, including its discovery, validation, performance evaluation and its comparison with existing scoring systems. The original dataset was randomly divided into a discovery and a validation cohort at a ratio of 7:3. In the discovery cohort, the SMOTE algorithm is used for sample balancing, and then models are built using SVM, RF, XGBoost and ANN models. The models are trained and tested using tenfold cross-validation to select the optimal model and perform feature importance analysis. Next, the predictive generalization and reliability of the model are validated in the validation cohort. The superior performance of the model was further validated by comparing its performance with the commonly used ISS, TI, and GCS scores in the validation cohort.

In this study, we constructed two separate ML models using the collected clinical data of polytrauma patients (Fig. 2 and Supplementary Figure S2). First, a polytrauma mortality model was developed to predict the deadly outcome of polytrauma patients upon hospital admission (Fig. 2). All eligible patients from 2020 to 2022 were randomly divided into a discovery cohort (70% of the original dataset) and a validation cohort (30% of the original dataset). The discovery cohort was used to train a ML model through supervised classification, while the validation cohort was used for external validation. The number of positive samples (deaths) is limited, resulting in a survival to death ratio of approximately 9:1 (478 alive, 51 deceased) in the development queue of two death risk prediction models. To address this imbalance, we utilized the Synthetic Minority Over-sampling Technique (SMOTE) algorithm to oversample the minority class samples in the two model development cohorts^25^. We generated synthetic samples in the feature space of minority class samples to adjust the positive-to-negative sample ratio to 2:1 (478 alive, 239 deceased). Subsequently, we used four ML algorithms, including RF, SVM, ANN and XGBoost, to build models for predicting mortality upon hospital admission for polytrauma patients. We tuned the model hyperparameters by defining a hyperparameter search space and conducting 50 trials within this space to find optimal values. Sampling and pruning algorithms were employed to efficiently determine the best parameter combination, ensuring optimal model performance and preventing overfitting. The optimized hyperparameter settings for each ML algorithm were listed in Supplementary Table S3. In the discovery cohorts, we employed tenfold cross-validation, dividing the data into 10 folds, with each fold serving as the test set while the remaining 9 folds as the training set. This process was repeated 10 times for training and testing. After the construction of four ML models using the discovery dataset, we also independently evaluated their performance using the validation dataset (Fig. 2).

Besides the mortality prediction model, we developed a prognostic model that predicts the complexity of polytrauma patients using the collected dataset as well (Supplementary Figure S2). To estimate the complexity of polytrauma patients, we used the staying time in ICU as the measurement and classified all 674 survived patients into mild, moderate and severe groups (Supplementary Figure S2). In this cohort, the male to female ratio was 1.76:1, and the average age was 54.53 years. Specifically, 375 polytrauma patients with ICU stays of 3 days or less were grouped into the mild group, while 129 patients with ICU stays of 8 days or more were grouped into the severe group. This resulted in a final dataset of 504 patients for complexity model construction (Supplementary Figure S2). Similarly, this cohort was further separated into a discovery dataset (361 patients in total, including 267 mild patients and 85 severe patients) and validation dataset (152 patients in total, including 108 mild patients and 44 severe patients) to develop and validate the prognostic model of polytrauma complexity upon hospital admission (Supplementary Figure S2). For the imbalance in the classification of mild and severe patients in the discovery dataset, we also used SMOTE to adjust the patient ratio from 3:1 (267 alive, 85 deceased) to 2:1 (267 alive, 133 deceased). In addition, we evaluated the performance of this complexity model and compared its performance with existing scoring systems (Supplementary Figure S2). Similarly, the optimized hyperparameter settings for each ML model are listed in Supplementary Table S4.

### ML algorithms

We used four different ML methods in developing the models, including RF, SVM, ANN and XGBoost. These methods had different underlying algorithms in training the model. The SVM model uses all the inputted clinical features and finds a hyperplane (Decision Boundary) that maximizes the gap between the two categories for classifying new instances^26^. By adjusting parameters to adapt to different data and problems, it maps the input data from low-dimensional space to high-dimensional space, making the data more linearly separable in high-dimensional space, and predicting the final classification. In the random forest model, all clinical features are used as the input data for the model. After a series of processing such as data preprocessing and standardization, these data form a set of feature vectors. The model trains on these features, and by constructing multiple decision trees and voting or averaging their prediction results, it obtains the final predicted classification result^27^. Each decision tree is trained on different parts of a dataset, which can reduce the variance of the model and improve the robustness and accuracy of the model. Similarly, XGBoost also uses all clinical features as input, processing them into a set of feature vectors. It is based on the gradient boosting framework, and by gradually adding new decision trees during the iterative process, it improves the predictive performance of the model^28^. In each round of iteration, XGBoost builds a new weak learner by focusing on the residuals of the previous round model, i.e., the difference between the predicted value and the actual value, thus capturing the complex relationships in the data more effectively. This process helps the model gradually reduce the error on the training data and improve generalization ability. The output result is made by training multiple weak learners and combining their prediction results to make the final prediction of the category situation. In contrast, the ANN is a deep learning model inspired by the structure of the human brain^29^. Our ANN model uses a multilayer perceptron classifier (MLPClassifier). The input of the model is all clinical features, which are passed to the neural network through the input layer. The neurons in the hidden layer learn the mapping relationship from input to output by adjusting the connection weights between nodes, such as the structure of the death prediction model is composed of a layer containing 10 neurons. The ReLU function is used as the activation function of the hidden layer, which introduces non-linearity and helps the model capture the complex relationships in the data. L2 regularization is introduced, and the alpha parameter is adjusted to control the strength of regularization, to penalize the square sum of the model’s weights, and prevent overfitting. The output layer uses the Sigmoid function as the activation function, mapping the output to between 0 and 1, predicting the death classification and complexity classification situation. The parameter settings of the polytrauma mortality risk or complexity model can be retrieved from Supplementary Tables S3 and S4, respectively.

### Model evaluation

To evaluate the performance of the constructed ML models, we calculated the metrics of each model in the discovery and validation datasets, respectively (Fig. 2 and Supplementary Figure S2). The performance metrics include *Accuracy*, *Recall*, *F-*score and the *AUC* value (see Supplementary Text for the details). The performance of each model in the discovery cohort was assessed by averaging the metrics at each round of tenfold validation. To evaluate the model’s performance against three commonly used scoring systems, we also compared the performance metrics of ML models with those of ISS, TI and GCS. In addition, we performed the feature analysis to elucidate the features that contributed mostly to the outcome prediction in the model.

### Model benchmark

We implemented four ML algorithms (SVM, RF, XGBoost and ANN) using *Python* (version 3.7) and *scikit*-*learn* (version 1.0.2). The sample balance was achieved by employing the oversampling method in *imblearn*^30^. Model parameters for each model were automatically optimized by *Optuna*^31^. Model evaluation, model comparison, and visualization of the feature importance were conducted using the *matplotlib* library (version 3.5.3).

## Results

### Mortality risk prediction model

#### The development and validation of the mortality risk prediction model

**Figure 3:**
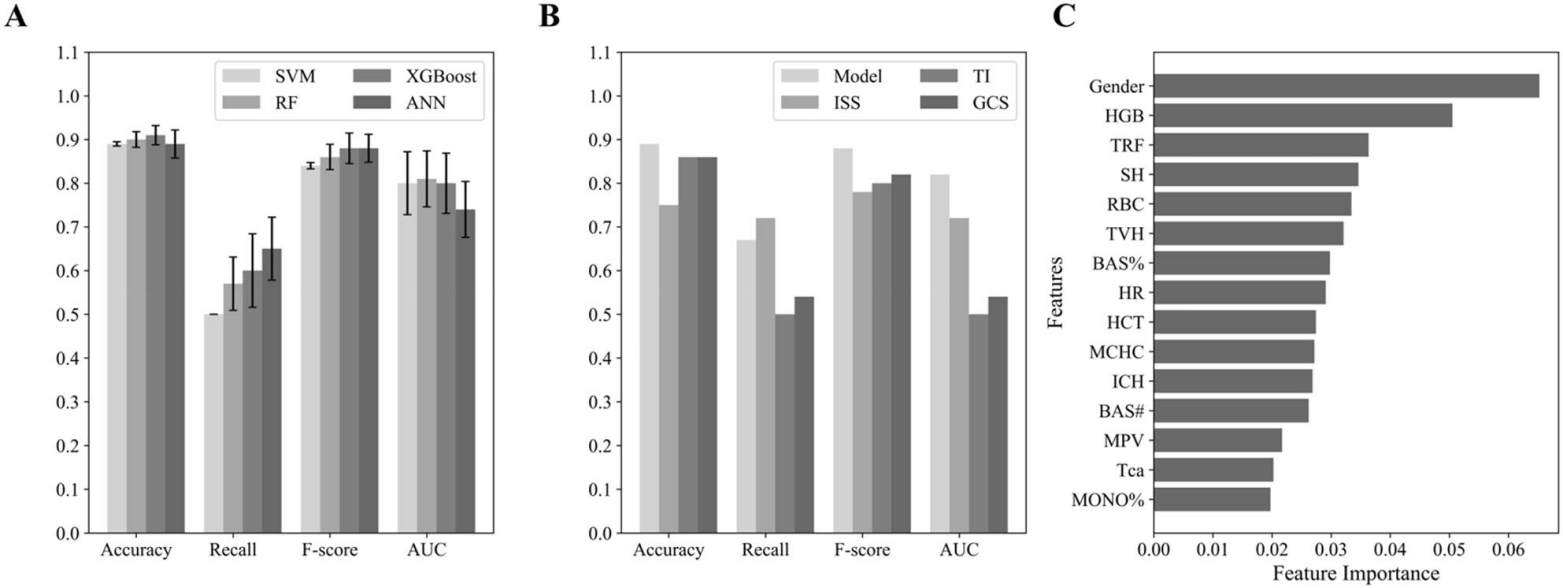
The performance metrics of the mortality prediction models. (**A**) The mean value and the standard deviation of the performance indicators, including *Accuracy*, *Recall*, *F*-score and *AUC*, of four ML models (SVM, RF, XGBoost and ANN) using ten-fold cross-validation was shown as the bar chart. **(B)** The performance indicators of the optimal ML model for predicting the mortality risk in the validation cohort were compared against the commonly used severity scores, including ISS, TI and GCS. **(C)** The top 15 features that contribute to the polytrauma mortality model classification were shown. HGB, Hemoglobin; TRF, Thoracic rib fracture; SH, Superficial hematoma; RBC, Red blood cell count; TVH, Thoracic visceral hematoma; BAS%, Basophil percentage; HR, Heart rate; HCT, Hematocrit; MCHC, Mean corpuscular hemoglobin concentration; BAS#, Basophil count; MPV, Mean platelet volume; Tca, Total calcium; MONO%, Monocyte percentage.

First, we developed a mortality risk prediction model for 756 selected polytrauma patients using four ML algorithms, including SVM, RF, XGBoost and ANN. We utilized ten-fold cross-validation to assess the performance of each model (Fig. 3A). The XGBoost model showed the best overall predictive performance among the four ML models. While its *AUC* value (0.80) was slightly lower than that of the SVM (0.804) and RF (0.82) models, it had higher overall performance (*Accuracy* = 0.91, *Recall* = 0.60, and *F-*score = 0.88) than that of SVM (*Accuracy* = 0.89, *Recall* = 0.5, and *F-*score = 0.84) and RF (*Accuracy* = 0.90, *Recall* = 0.57, and *F-*score = 0.86) models. Although the ANN model showed a slightly higher *Recall* value (0.65) than that of other models, its *AUC* value (0.74) was much smaller. Therefore, we chose the XGBoost model as the polytrauma mortality risk prediction model for further analysis. In the validation dataset, we confirmed the good performance of this XGBoost polytrauma mortality prediction model (Fig. 3B). The *Accuracy* value and *F-*score was 0.89 and 0.88 respectively, while the recall value was 0.68. Additionally, the *AUC* had a value at 0.82. These results indicated the XGBoost model’s good generalization and reliability in an independent validation dataset, suggesting its potential in clinical applications.

#### Comparison of the optimal mortality risk prediction model with common scoring systems

We also evaluated the performance of XGBoost polytrauma mortality prediction model against current clinical measurements by comparing the performance metrics of the XGBoost model with three commonly used scoring systems, including ISS, TI and GCS (Fig. 3B). We observed that the XGBoost polytrauma mortality prediction model significantly outperformed TI and GCS on all four metrics (Fig. 3B). Although the recall rate of ISS was higher than the XGBoost model, its *Accuracy, F-score* and *AUC* were lower (Fig. 3B). Therefore, the traditional scoring systems may have limited ability in predicting the mortality risk of polytrauma patients. In contrast, the XGBoost model has higher accuracy and balance in predicting the mortality risk of polytrauma patients. This suggests that the XGBoost model may distinguish dead and alive polytrauma patients early, while covering a higher proportion of the dead patients.

### Polytrauma complexity prediction model

#### The development and validation of the complexity prediction model

**Figure 4:**
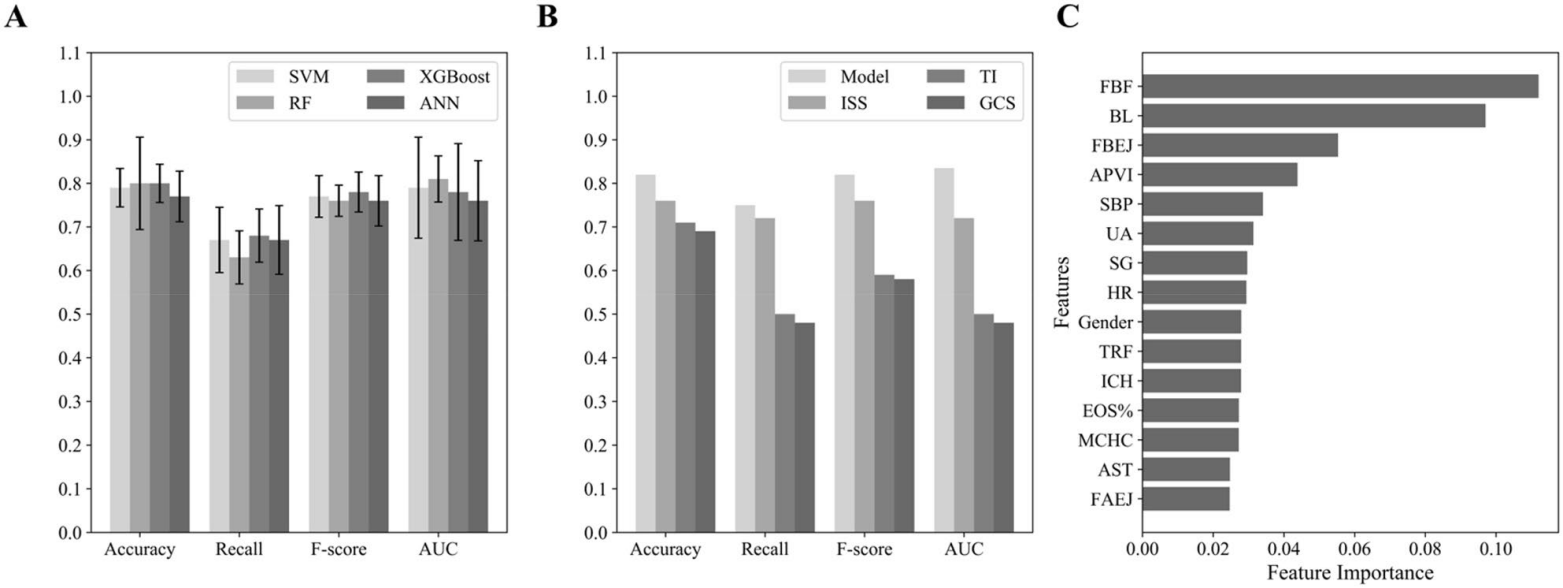
The performance metrics of the complexity prediction models for polytrauma patients upon ICU admission. (**A**) The bar chart of the mean and standard deviation of the performance metrics (*Accuracy*, *Recall*, *F*-score, *AUC*) for predicting the complexity of polytrauma patients upon ICU admission using four machine learning models (SVM, RF, XGBoost, ANN) in the discovery cohort was shown. **(B)** The performance indicators of the optimal model for predicting ICU admission complexity in the validation cohort was compared against the commonly used scores, including ISS, TI, GCS. **(C)** The top 15 features that contribute to the predictive model of the complexity of polytrauma patients upon ICU admission were shown. FBF, Facial bone fracture; BL, Blood loss; FBEJ, Fracture below elbow joint; APVI, Abdominopelvic visceral injury; SBP, Systolic blood pressure; UA, Uric Acid; SG, Specific Gravity; HR, Heart Rate; TRF, Thoracic rib fracture; ICH, Intracranial hematoma; EOS%, Eosinophil percentage; MCHC, Mean corpuscular hemoglobin concentration; AST, Aspartate aminotransferase; Fracture above elbow joint (FAEJ).

Next, we developed a complexity risk prediction model to predict the complexity upon hospital admission using four ML algorithms (Supplementary Figure S2), and calculated the performance metrics for four polytrauma complexity prediction models (Fig. 4A). Among them, the RF model (*Accuracy* = 0.8, *AUC* = 0.81) exhibited slightly higher *Accuracy* and *AUC* compared to the XGBoost model (*Accuracy* = 0.8, *AUC* = 0.78), though its *Recall* value and *F-*score ranked the lowest among the four models. Although the *AUC* value (0.79) of the SVM model is slightly higher than that of the XGBoost model, the other performances (*Accuracy* = 0.79, *Recall* = 0.67, *F-score* = 0.77) are much lower than the XGBoost model. The ANN model’s performance (*Accuracy* = 0.77, *Recall* = 0.67, *F-score* = 0.76, *AUC* = 0.76) are far lower than the XGBoost model. Therefore, the XGBoost model, which can learn meaningful signals from complex features, showed the best overall predictive performance among the four models in predicting polytrauma complexity. In the validation dataset, the XGBoost model showed good predictive performance for polytrauma complexity as well, with the *Accuracy*, *Recall*, *F-*score and *AUC* values at 0.82, 0.75, 0.82 and 0.83, respectively (Fig. 4B). The consistent performance on the external validation dataset confirmed the XGBoost model's generalization ability in predicting the complexity of polytrauma patients.

#### Comparison of optimal complexity prediction model with common scoring systems

In addition, we compared our XGBoost polytrauma complexity model with three commonly used scoring systems (ISS, TI, GCS) in predicting the patient’s complexity after hospital admission using the validation dataset (Fig. 4B). The performance metrics of the XGBoost model were higher than all the three existing severity scores, including ISS (*Accuracy* = 0.76, *Recall* = 0.72, *F*-*score* = 0.76), TI (*Accuracy* = 0.71, *Recall* = 0.5, *F*-*score* = 0.59), and GCS (*Accuracy* = 0.69, *Recall* = 0.48, *F*-*score* = 0.58). Moreover, the *AUC* value of our XGBoost model was higher than those of the ISS (0.72), TI (0.5) and GCS (0.48). These results suggest that the XGBoost model had better predictive performance than the currently used clinical severity scores in predicting the complexity of polytrauma patients as well.

### Feature importance analysis

To understand the reason why the ML models have better performance than existing scoring systems (Figs. 3B and 4B), we performed the feature importance analysis for both XGBoost prognostic models (Figs. 3C and 4C). We first explored the features influencing the mortality risk of polytrauma patients and ranked them based on their contribution to the model (Fig. 3C). The key predictive factors for polytrauma mortality are closely aligned with those used in commonly used scoring systems. For example, thoracic rib fracture (TRF), thoracic visceral hematoma (TVH) and superficial hematoma (SH) are among the top-ranked influencing factors. These features are all factors related to the extent and complexity of the injury site. Additionally, we identified several factors like gender, heart rate (HR), and various blood test indicators. Notably, blood test indicators ranked at the top among the 15 most influential features, underscoring their significance in improving prediction accuracy and reliability (Fig. 3C). These indicators are usually not taken into account by traditional scoring systems.

Similarly, we performed the feature importance analysis for our XGBoost disease complexity model as well, and explored the top 15 ranked contributing features (Fig. 4C). Among them, facial bone fracture (FBF) and blood loss emerged as the top factors influencing the polytrauma complexity, followed by articular fractures, and visceral injuries. This highlighted their significance in predicting the trauma complexity. Additionally, several laboratory tests, such as uric acid (UA), specific Gravity (SG), aspartate aminotransferase (AST), eosinophil percentage (EOS%), mean corpuscular hemoglobin concentration (MCHC) and systolic blood pressure (SBP), played an important role in predicting the disease complexity. These features reflect some aspects of metabolism, liver and kidney function and blood status that are important in polytrauma outcomes, although they may be often ignored in all three traditional scoring systems.

## Discussion

In this study, we developed two ML models to predict the mortality risk and complexity of polytrauma patients after hospital admission using clinical diagnoses and physiological parameters. We collected real-world data from a cohort of 756 polytrauma patients for training and validating these two ML models, and evaluated their performances. Our results suggested that XGBoost models are optimal ML models in predicting both the mortality risk and complexity of polytrauma patients (Figs. 3A and 4A), which had good performance in the training dataset and kept good capabilities in the separated validation dataset (Figs. 3B and 4B). Furthermore, our ML models outperformed several traditional scoring systems (Figs. 3B and 4B), underscored the advantage of XGBoost models in mining data with complex features, effectively capturing data relationships, and improving the assessment accuracy of polytrauma mortality and complexity.

In current clinical practices, several scores were used to estimate the severity of trauma patients. First, the TI evaluates the severity of trauma using five direct clinical parameters, including injury location, injury type, circulation, respiration and consciousness^10^. This allows the prognostic prediction of trauma patients at the prehospital stage. Second, the ISS is a score used for rapidly assessing the severity of injuries in patients. It involves dividing the patient's body into six different regions: head and neck, trunk (including neck, chest, and abdomen), face, limbs, external genitalia, and pelvis. Each region is then assigned a score based on the Abbreviated Injury Scale (AIS)^32^. These scores are then weighted and combined to generate an overall score. The ISS is primarily utilized in trauma centers to aid healthcare professionals in quickly assessing the severity and urgency of injuries in patients, enabling appropriate treatment measures to be taken. The proportion of patients completing ISS scores within 30 min has become an important quality control index for trauma centers in China. Third, the GCS score is used to assess the level of consciousness in patients^11^. It primarily evaluates a patient's level of consciousness through assessing their eye response, verbal response, and motor response. The GCS is mainly applied in trauma centers to evaluate the severity of brain function injuries. In the process of polytrauma patients, GCS score can not only directly reflect the degree of craniocerebral injury, but also serve as an indirect indicator to reflect the severity of patients with multiple injuries. Although these three scores had some differences in their calculation and clinical application, the parameters used for calculation of all three scores were easy to get at the first presentation of trauma patients. In our models, we used a variety of clinical information as the input features in predicting polytrauma severity, including several physiological parameters and laboratory test results. Exploring the contribution of features to polytrauma mortality and complexity prediction, we observed some factors had significant roles in both models, including gender, fractures, visceral injury and ICH (Figs. 3C and 4C). These may be the major factors that are correlated to trauma complexity and mortality. Our results highlighted the influential factor of some physiological and biochemical indicators in trauma complexity and severity, such as the gender and hemoglobin levels. This is consistent to the suggestions made in several previsou studies^33–35^. For example, Staudenmayer et al*.*^33^ observed the important roles of gender and HR in trauma outcomes. Da Costa et al*.*^34^ and Napolitano et al*.*^35^ also found significant implications of RBC and hemoglobin in trauma. Therefore, introducing these indicators in the ML prognostic models should increase their performance in predicting the mortality and complexity of polytrauma patients. This may partially explain why our ML models had better performance than ISS, TI and GCS in predicting the severity or complexity of polytrauma patients (Figs. 3B and 4B).

Several existing ML models have been developed to predict the mortality of trauma patients^19,36^. For example, Li et al*.*^37^ constructed an early warning score by multivariate logistic regression analysis on screened important features. Their study aims to apply the developed models for early screening of high-risk patients, establish an early warning system for mortality risk stratification, and subsequently implement early interventions to improve prognosis. Their mortality model showed a high *AUC* value at 0.941 in a smaller dataset (307 cases, 45 deaths), although they did not show its performance in an independent validation dataset. In another study, Zhang et al*.*^36^ developed mortality prediction models for traumatic shock patients using several ML algorithms, including decision trees, logistic regression and random forest, in a cohort of 281 patients. They constructed a random forest model as the optimal prognostic model, achieving an *AUC* value at 0.856. However, the model only achieved an AUC value of 0.741 in the independent validation dataset, indicating that its generalization ability needs to be improved. Both of these mortality risk model studies are primarily aimed at early screening of high-risk emergency trauma patients, establishing early warning of mortality risk for patients, and subsequently implementing early interventions to improve prognosis. Similarly, ML models have been developed to predict the disease severity for trauma patients as well^38,39^. For example, Van Rein et al*.*^38^ utilized eight predictors chosen based on clinical reasoning to develop and validate a prediction model for prehospital trauma triage, which can allocate patients to different trauma centers based on predicted severity. Instead, Staziaki et al*.*^39^ developed a SVM model to predict ICU admission and extended stay of length for torso trauma patients using clinical and imaging data. In contrast, our model is specifically designed for polytrauma patients, which are presumably the major type of severe trauma. Our models can predict both the mortality and the complexity for polytrauma patients when they are admitting to hospital. This may assist doctors in evaluating the mortality and severity of polytrauma patients at their early presentation, and then help them make suitable clinical intervention or coordinate the patient’s referral.

Although our ML models had good performance in predicting the severity and mortality of polytrauma patients, our study had some limitations. First, the training and validation datasets were collected from a single medical center, which may restrict the external validity of the model. Additionally, this single center study might also limit our choice of machine learning algorithms, thereby affecting the performance and generalizability of the model. Second, our study was a retrospective analysis using existing hospital records. This may introduce some biases due to the variability in the timing of laboratory tests and the vital signs of polytrauma patients. Third, the sample size of our cohort is relatively small from a machine learning perspective. This may limit the generalization ability of our model, facing challenges such as overfitting and difficulty in capturing complex relationships. To cope with these challenges, we adopted a cross-validation strategy by splitting the dataset multiple times, training and evaluating the model on different training and validation sets. This may help to reduce the risk of overfitting, improve the stability of the model performance evaluation, and help us to understand the model’s generalization performance. At the same time, cross-validation may overcome the biases of sample selection. In sum, although we have made every effort to avoid the potential shortcomings and our model performs well on the current dataset, its performance may decline on a larger or more diverse dataset. To overcome these limitations, future work should aim to expand the patient cohort in scale and scope to enhance the model’s universality and stability. A larger and multi-center prospective study is required to validate the performance of the prognostic models of polytrauma patients. Additional trauma outcome-relevant features, such as genetic, immunological and psychological factors, may help to improve model sophistication and accuracy. Some other ML models, such as deep learning and reinforcement learning algorithms, can be explored to enhance the model flexibility and performance. Furthermore, the randomized controlled trial (RCT) has been widely used as a research design for investigations of the clinical effectiveness of new medical interventions^40–42^. Randomized studies are conducted by randomly assigning experimental subjects or conditions, to control other variables that may affect the results, and to evaluate the effects of a certain intervention or treatment. A future RCT analysis may be performed to estimate the benefits of the clinical application of our ML models of polytrauma mortality and complexity.

In conclusion, the prognostic models we proposed here may offer physicians early prediction of the mortality and complexity of polytrauma patients, which has some potential values in aiding the clinical management of polytrauma patients.

### Supplementary Information

Supplementary Information.

## Data Availability

The clinical data that support the findings of our study were collected from the *Pizhou* People’s Hospital. Restrictions apply to the availability of these data, which were used for the current study. Therefore, the original data are not publicly available. However, data are available from the authors upon reasonable request and with permission of the Ethics Committee of *Pizhou* People’s Hospital. The corresponding author can be contacted for guidance concerning the data request.
