# Supplementary material for: Predicting the complexity and mortality of polytrauma patients with machine learning models
Source: Sci Rep. 2024 Apr 9;14:8302. doi: 10.1038/s41598-024-58830-0 (PMC11004111; doi:10.1038/s41598-024-58830-0)
Supplement: Supplementary file 1 — Supplementary Information. [file 41598_2024_58830_MOESM1_ESM.docx]

**Supplementary Files**

**Supplementary Text. The performance metrics used in model evaluation.**

**Supplementary Table S1. The demographic and baseline characteristics of collected clinical features.** A total of 70 clinical features were collected for polytrauma patients, including demographic information, vital signs, laboratory tests, and clinical diagnoses upon ICU admission by physicians. These features were collected the information of clinical outcome of each patient as well. For each patient, three existing trauma severity scores, including ISS, TI and GCS, were also calculated.

**Supplementary Table S2. The categorized primary diagnosis of polytrauma patients upon ICU admission.** The diagnosis at ICU admission includes various clinical diagnoses by physicians before data preprocessing. To normalize the primary diagnosis made by physicians, we refined the numerous clinical diagnoses of polytrauma patients using the rules in this table.

**Supplementary Table S3. The hyperparameter settings of four ML models (SVM, RF, XGBoost and ANN) in predicting the mortality risk of polytrauma patients.** To find the optimal hyperparameter combination, we performed 50 trials for each ML model and dynamically constructed the hyperparameter search space.

**Supplementary Table S4. The hyperparameter settings of four ML models (SVM, RF, XGBoost and ANN) in predicting the disease complexity of polytrauma patients.** To find the optimal hyperparameter combination, we performed 50 trials for each ML model and dynamically constructed the hyperparameter search space.

**Supplementary Figure S1.** The schematic flowchart of our study.

**Supplementary Figure S2.** **The construction of polytrauma complexity model, including its discovery, validation, performance evaluation and its comparison with existing scoring systems.** The original dataset was randomly divided into a discovery and a validation cohort at a ratio of 7:3. In the discovery cohort, the SMOTE algorithm is used for sample balancing, and then models are built using SVM, RF, XGBoost and ANN models. The models are trained and tested using 10-fold cross-validation to select the optimal model and perform feature importance analysis. Next, the predictive generalization and reliability of the model are validated in the validation cohort. The superior performance of the model was further validated by comparing its performance with the commonly used ISS, TI, and GCS scores in the validation cohort.

**Supplementary Text. The performance metrics used in model evaluation.**

To compare the performance between different ML models (SVM, RF, XGBoost, ANN), and between the optimal model and three scoring systems (ISS, TI and GCS), we calculated the following four metrics, including *Accuracy*, *Recall*, *F-score* and *AUC* value.

1. ***Accuracy***: This measures the proportion of correctly classified samples among all predicted samples, which was calculated as the formula below.

$$Accuracy=\frac{TP+TN}{TP+TN+FP+FN}$$

Here, $TP$ represents the number of samples in the positive class which were predicted as positive, while $TN$ represents the number of samples in the negative class which were predicted as negative. $FP$ represents the number of samples in the negative class which were predicted as positive, and $FN$ represents the number of samples in the positive class which were predicted as negative.

1. ***Recall***: This measures the proportion of positive samples that were correctly predicted. It was calculated as the formula below.

$$Recall=\frac{TP}{TP+FN}$$

2. ***F***-**score**: This calculates the harmonic mean of *Precision* and *Recall*. In this context, precision refers to the proportion of actual positive samples in the predicted positive samples. The precision and the *F*-score are calculated as the formulas below.

$$Precision=\frac{TP}{TP+FP}$$

3. ***Area Under Curve (AUC***): This measures the area under the Receiver Operating Characteristic (ROC) curve, which is enclosed by the ROC curve and the coordinate axis. *AUC* is the probability that a randomly chosen positive sample scores higher than a randomly chosen negative sample, which is calculated using the formula below.

$$AUC=\frac{\sum_{i} (p_{i},n_{j})_{p_{i}>n_{j}}}{(TP+FN)*(TN+FP)}$$

Here, $p_{i}$ is the predicted score of all samples in the positive class, and $n_{j}$​ is the predicted score of all samples in the negative class.

**Supplementary Table S1. The demographic and baseline characteristics of collected clinical features.** A total of 70 clinical features were collected for polytrauma patients, including demographic information, vital signs, laboratory tests, and clinical diagnoses upon ICU admission by physicians. These features were collected the information of clinical outcome of each patient as well. For each patient, three existing trauma severity scores, including ISS, TI and GCS, were also calculated.

| **Characteristic** | **Survival (n=674)** | | | | | | | | **Death (n=82)** | | |
| --- | --- | --- | --- | --- | --- | --- | --- | --- | --- | --- | --- |
|  | **Mild (n=375)**  ICU days<=3 | | **Severe (n=129)**  ICU days>=8 | | | **Total** | |  | | | |
| ***Demographics*** |  | | | |  | |  | | |  |
| Age (year) ^$^ | | 53.39±20.37 | | 57.22±17.03 | | 54.53±19.52 | | | 58.5±20.99 | | |
| Gender ^*^ | |  | |  | |  | | |  | | |
| Male | | 229(61.06) | | 94(72.86) | | 430(63.8) | | | 28(34.2) | | |
| Female | | 146(38.93) | | 35(27.13) | | 244(36.2) | | | 54(65.8) | | |
| ***Vital signs*** | |  | |  | |  | | |  | | |
| Body temperature (Temp)^$^ | | 36.50±0.20 | | 36.50±0.15 | | 36.51±0.21 | | | 36.44±0.21 | | |
| Systolic blood pressure (SBP) ^$^ | | 131.65±22.40 | | 121.90±26.51 | | 129.06±23.41 | | | 113.54±29.22 | | |
| Diastolic blood pressure (DBP) ^$^ | | 76.51±13.48 | | 72.03±14.93 | | 75.21±13.90 | | | 67.47±17.70 | | |
| Heart rate (HR) ^$^ | | 84.05±16.28 | | 93.03±20.80 | | 87.12±18.1 | | | 101.03±22.25 | | |
| Blood loss (BLV) ^$^ | | 339.79±319.12 | | 516.17±326.7 | | 366.42±306.15 | | | 546.27±310.27 | | |
| ***Laboratory tests*** | |  | |  | |  | | |  | | |
| White blood cell count (WBC) ^$^ | | 11.19±5.28 | | 11.82±6.50 | | 11.35±5.62 | | | 13.91±7.71 | | |
| Lymphocyte percentage (LYM%)^$^ | | 11.97±8.07 | | 13.69±10.74 | | 12.53±9.1 | | | 10.56±7.13 | | |
| Neutrophil percentage (NEU%)^$^ | | 81.25±10.32 | | 78.48±13.11 | | 80.38±11.48 | | | 83.69±8.24 | | |
| Monocyte percentage (MONO%)^$^ | | 5.88±2.41 | | 6.48±2.42 | | 6.03±2.4 | | | 5.09±1.95 | | |
| Eosinophil percentage (EOS%)^$^ | | 0.21±0.20 | | 0.21±0.20 | | 1.48±1.32 | | | 1.6±1.31 | | |
| Basophil percentage (BAS%)^$^ | | 0.20±0.19 | | 0.24±0.23 | | 0.22±0.21 | | | 0.16±0.16 | | |
| Lymphocyte count (LYM#)^$^ | | 1.13±0.69 | | 1.27±0.76 | | 1.16±0.72 | | | 1.39±1.08 | | |
| Neutrophil count (NEUT#)^$^ | | 9.37±4.98 | | 9.73±6.06 | | 9.46±5.32 | | | 11.78±6.79 | | |
| Monocyte count (MONO#)^$^ | | 0.61±0.30 | | 0.70±0.39 | | 0.63±0.32 | | | 0.68±0.42 | | |
| Eosinophil count (EOS#)^$^ | | 0.11±0.09 | | 0.09±0.07 | | 0.10±0.09 | | | 0.11±0.09 | | |
| Basophil count (BAS#)^$^ | | 0.02±0.01 | | 0.02±0.02 | | 0.02±0.02 | | | 0.02±0.02 | | |
| Red blood cell count (RBC)^$^ | | 4.00±0.62 | | 3.73±0.67 | | 3.94±0.64 | | | 3.23±0.78 | | |
| Hemoglobin (HGB)^$^ | | 124.06±19.93 | | 115.75±21.18 | | 121.95±20.32 | | | 99.63±24.00 | | |
| Hematocrit (HCT)^$^ | | 37.04±5.61 | | 34.70±6.04 | | 36.46±5.76 | | | 30.10±7.17 | | |
| Mean corpuscular volume (MCV)^$^ | | 92.66±5.68 | | 93.13±4.72 | | 92.65±5.33 | | | 93.36±4.86 | | |
| Mean corpuscular hemoglobin (MCH)^$^ | | 31.00±2.08 | | 31.02±1.57 | | 30.95±1.89 | | | 30.86±1.52 | | |
| Mean corpuscular hemoglobin  concentration (MCHC)^$^ | | 334.50±8.83 | | 333.13±9.11 | | 334.08±9.06 | | | 330.79±9.87 | | |
| Red cell distribution width-standard deviation (RDW-SD) ^$^ | | 42.66±3.65 | | 44.32±5.23 | | 43.014±4.23 | | | 44.55±4.69 | | |
| Platelet count (PLT)^$^ | | 205.97±70.73 | | 222.28±88.68 | | 209.58±77.98 | | | 173.5±81.05 | | |
| Mean platelet volume (MPV)^$^ | | 10.02±1.22 | | 10.10±1.39 | | 10.09±1.28 | | | 9.89±1.44 | | |
| Platelet distribution width (PDW)^$^ | | 11.19±5.28 | | 11.87±6.45 | | 11.34±5.60 | | | 13.50±7.53 | | |
| Ketone bodies (KET)^*^ | |  | |  | |  | | |  | | |
| Positive | | 221(58.93) | | 85(65.89) | | 393(58.4) | | | 44(53.6) | | |
| Negative | | 154(41.06) | | 44 (34.10) | | 281(41.6) | | | 38(47.4) | | |
| Urobilinogen (UBG)^*^ | |  | |  | |  | | |  | | |
| Positive | | 0(0.0) | | 0(0.0) | | 673(99.8) | | | 673(99.8) | | |
| Negative | | 375(100.0) | | 129 (100.0) | | 1(0.2) | | | 1(0.2) | | |
| Bilirubin (BIL)^*^ | |  | |  | |  | | |  | | |
| Positive | | 0(0.0) | | 7 (5.42) | | 665(98.66) | | | 81(98.78) | | |
| Negative | | 375(100.0) | | 122 (94.5) | | 9(1.34) | | | 1(1.22) | | |
| PH ^$^ | | 6.41±0.54 | | 6.23±0.48 | | 6.34±0.52 | | | 6.13±0.45 | | |
| Specific gravity (SG) ^$^ | | 1.07±1.09 | | 3.70±30.43 | | 1.56±13.33 | | | 1.02±0.006 | | |
| Uric acid (UA) ^$^ | | 290.88±92.46 | | 253.27±108.43 | | 283.75±102.20 | | | 313.48±119.68 | | |
| Blood urea nitrogen (BUN) ^$^ | | 6.12±1.84 | | 6.16±2.44 | | 283.75±99.5 | | | 7.42±2.57 | | |
| Total bilirubin (TBIL) ^$^ | | 16.24±7.88 | | 17.50±8.77 | | 16.43±8.01 | | | 18.87±16.10 | | |
| direct bilirubin (DBIL) ^$^ | | 3.328±1.93 | | 4.06±2.66 | | 3.49±2.18 | | | 5.18±3.16 | | |
| Total protein (TP) ^$^ | | 59.32±6.67 | | 58.26±7.35 | | 59.16±6.96 | | | 53.82±10.55 | | |
| Albumin (ALB) ^$^ | | 36.40±4.71 | | 35.64±7.29 | | 36.17±5.28 | | | 33.01±5.97 | | |
| Globulin (Glb)^$^ | | 22.83±3.87 | | 22.78±4.64 | | 22.91±4.26 | | | 20.79±6.21 | | |
| Alanine aminotransferase (ALT) ^$^ | | 26.81±17.23 | | 37.03±25.06 | | 30.14±20.35 | | | 33.03±20.34 | | |
| Aspartate aminotransferase (AST) ^$^ | | 33.44±20.41 | | 39.80±27.25 | | 34.92±22.59 | | | 42.93±23.01 | | |
| K^$^ | | 3.930±0.38 | | 4.05±0.77 | | 3.96±0.48 | | | 3.88±0.44 | | |
| Na^$^ | | 137.41±3.27 | | 135.47±15.21 | | 137.16±7.35 | | | 138.63±6.17 | | |
| Cl ^$^ | | 104.60±3.96 | | 102.96±7.33 | | 104.191±4.35 | | | 106.27±6.54 | | |
| Total calcium (Tca) ^$^ | | 2.22±1.82 | | 2.33±1.84 | | 2.21±1.49 | | | 2.04±1.49 | | |
| Fibrinogen (FIB) ^$^ | | 2.64±0.80 | | 3.02±1.16 | | 2.78±0.92 | | | 2.32±1.25 | | |
| ***Clinical diagnosis*** | |  | |  | |  | | |  | | |
| Circumferential skull fracture (CSF)^*^ | | 64(9.07) | | 34(5.60) | | 135(20.0) | | | 22(26.8) | | |
| Facial bone fracture (FBF)^*^ | | 34(9.07) | | 21(5.60) | | 74(10.9) | | | 11(13.4) | | |
| Cervical vertebra fracture (CVF)^*^ | | 2(0.53) | | 1(0.27) | | 3(0.4) | | | 2(2.4) | | |
| Lumbosacral vertebra fracture (LVF)^*^ | | 85(22.67) | | 42(11.20) | | 175(25.9) | | | 16(19.5) | | |
| Thoracic rib fracture (TRF)^*^ | | 144(38.40) | | 86(22.93) | | 318(47.1) | | | 44(53.6) | | |
| Pelvic fracture (PF)^*^ | | 45(12.00) | | 26(6.93) | | 103(15.2) | | | 12(14.6) | | |
| Fracture above knee joint (FAKJ)^*^ | | 3(0.80) | | 20(5.33) | | 32(4.7) | | | 13(15.8) | | |
| Fracture below knee joint (FBKJ)^*^ | | 49(13.07) | | 29(7.73) | | 114(16.9) | | | 18(21.9) | | |
| Fracture above elbow joint (FAEJ)^*^ | | 53(14.13) | | 45(12.00) | | 137(20.3) | | | 15(18.2) | | |
| Fracture below elbow joint (FBEJ)^*^ | | 24(6.40) | | 14(3.73) | | 58(8.6) | | | 6(7.3) | | |
| General joint dislocation (GJD)^*^ | | 10(2.67) | | 17(4.53) | | 36(5.3) | | | 2(2.4) | | |
| Vital joint dislocation (VJD)^*^ | | 35(1.60) | | 23(0.27) | | 7(1.0) | | | 2(2.4) | | |
| Circumferential brain injury (CBI)^*^ | | 64(9.33) | | 34(6.13) | | 91(13.5) | | | 21(25.6) | | |
| Spinal cord injury (SCI)^*^ | | 1(0.27) | | 2(0.53) | | 4(0.5) | | | 0(0.0) | | |
| Peripheral nerve injury (PNI)^*^ | | 12(3.20) | | 5(1.33) | | 20(2.9) | | | 3(3.6) | | |
| Thoracic visceral injury (TVI)^*^ | | 34(9.07) | | 31(8.27) | | 94(13.9) | | | 5(6.1) | | |
| Abdominopelvic visceral injury (APVI)^*^ | | 25(6.67) | | 32(8.53) | | 80(11.8) | | | 3(3.6) | | |
| Major vascular injury (MVI)^*^ | | 1(0.27) | | 2(0.53) | | 4(0.5) | | | 2(2.4) | | |
| Thoracic visceral hematoma (TVH)^*^ | | 126(33.60) | | 63(16.80) | | 252(37.3) | | | 31(37.8) | | |
| Abdominopelvic visceral  hematoma (APVH)^*^ | | 17(4.53) | | 10(2.67) | | 38(5.6) | | | 5(6.1) | | |
| Intracranial hematoma (ICH)^*^ | | 70(18.67) | | 50(13.33) | | 159(23.5) | | | 38(46.3) | | |
| Superficial hematoma (SH)^*^ | | 145(38.67) | | 52(13.87) | | 258(38.2) | | | 25(30.4) | | |
| ***Clinical Outcome*** |  | | | |  | |  | | |  |
| ICU length of stay (ICU LOS)^$^ | | 1.84±0.78 | | 13.39±8.19 | | 6.03±5.59 | | | 7.96±5.63 | | |
| ***Common scoring systems*** | | | | | | | | | | | |
| Injury Severity Score (ISS) ^$^ | | 17.08±8.70 | | 27.0±11.82 | | 19.87±10.12 | | | 26.30±12.07 | | |
| Trauma Index (TI)^$^ | | 14.376±2.23 | | 16.03±3.04 | | 14.77±2.54 | | | 16.91±3.32 | | |
| Glasgow Coma Scale (GCS)^$^ | | 14.304±2.03 | | 12.45±3.65 | | 13.97±2.37 | | | 11.17±4.79 | | |

^*^ For categorical features, the table shows the number (percentage) of patients in the class; ^$^ for continuous features, the table shows the mean value ± standard deviation of this feature of all patients in the class.

**Supplementary Table S2. The categorized primary diagnosis of polytrauma patients upon ICU admission.** The diagnosis at ICU admission includes various clinical diagnoses by physicians before data preprocessing. To normalize the primary diagnosis made by physicians, we refined the numerous clinical diagnoses of polytrauma patients using the rules in this table.

| **Categorized diagnosis** | **Specific diagnosis before preprocessing** |
| --- | --- |
| Circumferential skull fracture (CSF) | Intracranial emphysema, Intracranial air, Depressed skull fracture, Skull base fracture, Fracture of skull base, Depressed fracture of skull, Cerebrospinal fluid rhinorrhea, Temporal bone fracture, Occipital bone fracture, Sphenoid bone fracture, Parietal bone fracture, Frontal bone fracture |
| Facial bone fracture (FBF) | Alveolar Bone Fracture, Jaw Fracture, Zygomatic Fracture, Nasal Fracture, Facial Bone Fracture, Maxillary Sinus Fracture, Multiple Maxillofacial Fractures |
| Cervical vertebra fracture (CVF) | Fracture of Odontoid Process of Vertebra Dentata, Axis Fracture, Superior margin fracture of atlas, Cervical Spine Fracture |
| Lumbosacral vertebra fracture (LVF) | Multiple Thoracic Vertebral Fractures, Thoracic Spinous Process Fracture, Thoracic Vertebral Fracture, T4-T7 Thoracic Spinous Process Fractures |
| Thoracic rib fracture (TRF) | Lumbar Sacral Vertebral Fracture, Lumbar Sacral Vertebral Arch Fracture, Lumbar Vertebral Fracture, Lumbar Spondylolysis, L5 Lumbar Spondylolysis, Compression Fracture, Lumbar Sacral Spinous Process Fracture, Coccyx Fracture, Sacral Fracture |
| Pelvic fracture (PF) | Rib Fracture, Sternum Fracture |
| Fracture above knee joint (FAKJ) | Ischial Fracture, Pubic Fracture, Iliac Fracture, Acetabular Fracture, Pelvic Fracture, Dislocation of Sacroiliac and Sacrococcygeal Joint, Sacroiliac Joint Dislocation |
| Fracture below knee joint (FBKJ) | Femoral Shaft Fracture, Femoral Fracture, Slipped Epiphysis, Femoral Head Fracture, Old Intertrochanteric Fracture of the Femur, Subcapital Fracture of the Femur, Intertrochanteric Fracture of the Femur, Femoral Head Fracture, Femoral Neck Fracture, Lesser Trochanteric Fracture of the Femur |
| Fracture above elbow joint (FAEJ) | Coronoid fracture, Elbow fracture, Intercondylar humerus fracture, Condylar fracture, Patella fracture, Fibula fracture, Tibia fracture, Ankle bone fracture, Talus bone fracture, Ankle joint fracture, Ankle bone fracture, Big toe bone fracture, Heel bone fracture, Toe bone fracture, Metatarsal bone fracture, Cuboid bone fracture, Foot bone fracture |
| Fracture below elbow joint (FBEJ) | Scapular acromion process fracture, Clavicle fracture, Glenoid cavity fracture, Scapula fracture, Humerus head fracture, Shoulder peak bone fracture, Nodular bone fracture, Humerus bone fracture |
| General joint dislocation (GJD) | Olecranon Fracture, Elbow Joint Fracture, Intercondylar Humerus Fracture, Condylar Fracture, Forearm Fracture, Ulna Fracture, Radial Fracture, Triquetral Fracture, Thumb Fracture, Multiple Fractures of the Carpal Bones, Phalangeal Fracture, Scaphoid Fracture, Metacarpal Fracture, Hand Fracture, Pisiform Bone Fracture, Carpal Bone Fracture |
| Vital joint dislocation (VJD) | Dislocation of shoulder, Acromioclavicular joint dislocation, Second toe phalangeal dislocation, Sternoclavicular joint dislocation, Hip joint subluxation, Lower limb joint and ligament dislocation, Sprain and strain, Hip dislocation, Hip joint dislocation, Posterior hip joint dislocation, Elbow joint dislocation, Inferior radioulnar joint dislocation, Open ankle joint dislocation, Ankle joint dislocation, Carpal dislocation |
| Circumferential brain injury (CBI) | Atlantoaxial dislocation, Subluxation of atlantoaxial joint |
| Spinal cord injury (SCI) | Diffuse axonal injury, Traumatic brain injury, Cerebral contusion, Brain contusion and laceration, Concussion, Brain edema, Brainstem injury, Tentorial notch herniation, Brain herniation, Brainstem contusion |
| Peripheral nerve injury (PNI) | Lumbar spinal cord edema, Cervical spinal cord edema, Spinal cord injury, Traumatic paraplegia, Acute paraplegia |
| Thoracic visceral injury (TVI) | Nerve injury, Brachial plexus injury, Optic nerve injury, Common peroneal nerve palsy, Ulnar nerve damage, Radial nerve damage |
| Abdominopelvic visceral injury (APVI) | Thoracic esophageal injury, Lung injury, Pulmonary contusion, Acute pulmonary edema, Traumatic pericardial rupture, Pulmonary atelectasis, Atelectasis |
| Major vascular injury (MVI) | Liver injury, Liver contusion, Traumatic liver rupture, Ileum injury, Rectal injury, Traumatic diaphragmatic rupture, Bladder injury, Traumatic ileum rupture, Intestinal rupture, Traumatic intestinal rupture, Mesenteric injury, Mesenteric laceration, Mesenteric hemorrhage, Splenic injury, Traumatic splenic rupture, Gastric injury, Adrenal injury, Gastrointestinal bleeding, Renal pedicle injury, Renal injury, Renal contusion, Traumatic renal rupture, Bile duct injury, Pancreatic injury, Pancreatic injury, Traumatic rupture of the pancreatic tail, severe acute traumatic pancreatitis, mild acute traumatic pancreatitis |
| Thoracic visceral hematoma (TVH) | Lower leg great saphenous vein injury, Abdominal aortic injury, Aortic rupture, Internal jugular vein injury, Femoral artery injury |
| Abdominopelvic visceral hematoma (APVH) | Pleural effusion, Traumatic pleural effusion, pneumothorax, Traumatic pneumothorax, tension pneumothorax, Hydropneumothorax, Hemopneumothorax, Hemothorax |
| Intracranial hematoma (ICH) | Retroperitoneal hematoma, Intraperitoneal hemorrhage, Renal hematoma, Traumatic perirenal hematoma, Renal hemorrhage, Adrenal hemorrhage, Adrenal hematoma |
| Superficial hematoma (SH) | Basal ganglia hemorrhage, Cerebral hemorrhage, Traumatic intracranial hematoma, Epidural hematoma, Temporal epidural hemorrhage, Intracerebral hemorrhage, Intraventricular hemorrhage, Traumatic subdural hemorrhage, Frontotemporal acute subdural hemorrhage, Traumatic cerebral contusion, Subdural hematoma, Subdural hygroma, front temporoparietal subdural hygroma, Traumatic subarachnoid hemorrhage, Traumatic subarachnoid bleeding, Suprasellar cistern traumatic subarachnoid bleeding, Occipital lobe traumatic subarachnoid hemorrhage, Vertex traumatic subarachnoid hemorrhage, Subarachnoid hemorrhage, Traumatic subarachnoid hemorrhage |
| Circumferential skull fracture (CSF) | Aural hemorrhage, Oral mucosal bleeding, epistaxis, Traumatic sinus hematoma, Eyelid hematoma, Frontal hematoma, Front temporoparietal scalp hematoma, Lower leg hematoma, Thigh hematoma, Chest and back superficial body hematoma, Paraspinal muscle hematoma, Traumatic iliopsoas muscle hematoma, Subcutaneous hematoma |

**Supplementary Table S3. The hyperparameter settings of four ML models (SVM, RF, XGBoost and ANN) in predicting the mortality risk of polytrauma patients.** To find the optimal hyperparameter combination, we performed 50 trials for each ML model and dynamically constructed the hyperparameter search space.

| **Model** | **Hyperparameter Tuning** |
| --- | --- |
| SVM | Kernel=’linear’, C=0.0004538, gamma=0.5992, decision_function_shape=’ovr’ |
| RF | n_estimators=140, max_depth=4, random_state=111, min_samples_split=6 |
| XGBoost | max_depth= 3, learning_rate= 0.041499254984206445, n_estimators= 110, nthread=4, colsample_bytree= 0.5852576202921144 |
| ANN | hidden_layer_sizes=(10,), alpha= 0.0001435271433817171, activation=’tanh’ |

**Supplementary Table S4. The hyperparameter settings of four ML models (SVM, RF, XGBoost and ANN) in predicting the disease complexity of polytrauma patients.** To find the optimal hyperparameter combination, we performed 50 trials for each ML model and dynamically constructed the hyperparameter search space.

| Model | Hyperparameter Tuning |
| --- | --- |
| SVM | kernel=’linear’, C=0.1, gamma=6, decision_function_shape=’ovr’ |
| RF | n_estimators=373, max_depth=10, min_samples_split=5 |
| XGBoost | max_depth=5, learning_rate= 0.5941600723055731, n_estimators=414, nthread=4, colsample_bytree= 0.8791802192543063 |
| ANN | hidden_layer_sizes=(100,50), alpha= 1.0838208915741297e-06, activation=’relu’ |





**Supplementary Figure S1.** The schematic flowchart of our study.





**Supplementary Figure S2.** **The construction of polytrauma complexity model, including its discovery, validation, performance evaluation and its comparison with existing scoring systems.** The original dataset was randomly divided into a discovery and a validation cohort at a ratio of 7:3. In the discovery cohort, the SMOTE algorithm is used for sample balancing, and then models are built using SVM, RF, XGBoost and ANN models. The models are trained and tested using 10-fold cross-validation to select the optimal model and perform feature importance analysis. Next, the predictive generalization and reliability of the model are validated in the validation cohort. The superior performance of the model was further validated by comparing its performance with the commonly used ISS, TI, and GCS scores in the validation cohort.
